# Biased feedback in brain-computer interfaces

**DOI:** 10.1186/1743-0003-7-34

**Published:** 2010-07-27

**Authors:** Álvaro Barbero, Moritz Grosse-Wentrup

**Affiliations:** 1 Universidad Autónoma de Madrid (Departamento de Ingeniería Informática) and Instituto de Ingeniería del Conocimiento, Francisco Tomás y Valiente 11, 28049, Madrid, Spain; 2 Max Planck Institute for Biological Cybernetics, Spemannstr. 38, 72076 Tübingen, Germany

## Abstract

Even though feedback is considered to play an important role in learning how to operate a brain-computer interface (BCI), to date no significant influence of feedback design on BCI-performance has been reported in literature. In this work, we adapt a standard motor-imagery BCI-paradigm to study how BCI-performance is affected by biasing the belief subjects have on their level of control over the BCI system. Our findings indicate that subjects already capable of operating a BCI are impeded by inaccurate feedback, while subjects normally performing on or close to chance level may actually benefit from an incorrect belief on their performance level. Our results imply that optimal feedback design in BCIs should take into account a subject's current skill level.

## Findings

Brain-computer interfaces (BCIs) enable subjects to communicate without using the peripheral nervous system by recording brain signals and translating these into control commands [1]. To operate a BCI, subjects need to learn how to intentionally modulate certain characteristics of their brain signals in order to express their intention. For example, in motor imagery, one of the most frequently used experimental paradigms in BCIs [2], subjects are instructed to haptically imagine movements of either the left or right hand, which typically induces a decrease in power of the electromagnetic field of the brain over contralateral sensorimotor cortex in the *μ*- and *β*-frequency ranges (roughly 10-14 Hz and 20-30 Hz, respectively) [3]. The observed lateralization of this sensorimotor-rhythm (SMR) can then be used to infer a subject's intention.

As in any form of skill acquisition, subjects require feedback on their performance in order to learn how to optimally regulate their brain signals. While the importance of feedback in BCIs has long been recognized [1], surprisingly little is known on how feedback should be designed in BCIs in order to facilitate the skill acquisition process. In [4], the authors investigated whether instantaneous or delayed feedback proved to be more beneficial. While individual differences could be found, on average no significant effect was observed. Recently, the influence of realistic vs. abstract feedback on BCI performance was investigated [5]. However, the authors again found no evidence for a significant influence of the type of feedback on BCI performance. As such, it appears that the specfic feedback design has little influence on BCI performance.

It should be noted, however, that in previous studies only accurate feedback was considered. While it is generally accepted that feedback in skill acquisition should be timely and precise, motivation is also known to play an important role in BCIs (cf. [6]). Accordingly, subjects may benefit from feedback that trades feedback accuracy for motivation, e.g., by artificially biasing the belief subjects have on their success in the skill acquisition process.

**Figure 1:**
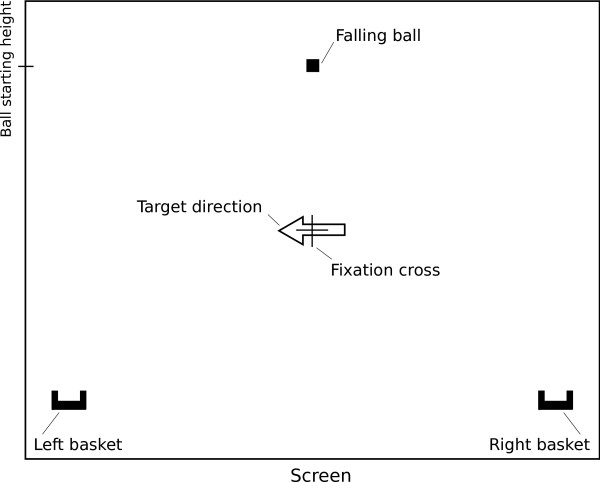
**Setup of visual feedback**. Arrangement of the elements present in the visual feedback interface of the used BCI system. The subject is told to look at the fixation cross, which is always present on the screen. During each trial an arrow showing the objective basket appears on screen. The position of the baskets is fixed, and the falling ball always starts at the shown position at the beginning of each trial.

In this work, we investigate the influence of such a feedback bias on BCI performance. Subjects participated in a standard BCI experiment, in which they were asked to navigate a falling ball into a basket in either the left or right corner of the screen by performing haptic motor imagery of either the left or right hand. A depiction of the visual interface is shown in Figure 1. Each experimental trial lasted four seconds, and was considered successful if the ball ended up in the correct half-side of the screen. While usually the horizontal position of the ball on the screen reflects the belief of the BCI system on a subject's intention, we artificially distorted this feedback. Specifically, every two milliseconds we coded the classifier's belief on a subject's intention as a value in the range [0-1]. Then, we drew a sample from a Gaussian distribution, and added this to the classifier's belief. The mean of this sample was chosen as a function of the type of bias, and its variance was determined heuristically and identical for all type of feedback to prevent subjects' awareness of the feedback bias (*σ*^2 ^= 3·10^-4^). If the resulting value was found to be larger/smaller than the current horizontal position of the ball (0/1 representing the left/right border of the screen), the ball was was moved one step (0.003 times the width of the screen) to the right/left. At the beginning of each trial, we pseudo-randomly chose one of five means for this random distortion, such that without any meaningful BCI control by the subject the falling ball would on average end up in 1.) the intended corner of the screen (strong positive bias), 2.) half-way between the center of the screen and the intended corner (weak positive bias), 3.) in the center of the screen (no bias), 4.) halfway between the center of the screen and the incorrect corner (weak negative bias), or 5.) in the incorrect corner (strong negative bias). As such, in 80% of the trials we biased the belief the subject had on her/his performance in either a positive or negative manner, while in the remaining 20% of trials subjects received accurate feedback.

Eleven healthy subjects with a mean age of 26.18 ± 4.14 years, seven of them male and four female, participated in the study, all except one were naive to BCIs. Every subject initially performed one session. Four subjects attaining a good level of BCI-control were asked to perform two additional sessions each, as we expected effects to be most prominent in well-performing subjects. Each session consisted of nine runs, with each run being composed of 15 trials per condition in pseudo-randomized order. The first three runs of each session, during which no feedback was presented to the subject, were used to train the classification system. During the following six runs, biased feedback was presented as discussed above. For each session, this resulted in a total of 36 trials for each of the five feedback biases. Mean classification accuracy was then computed for each session and feedback bias, using the undistorted classifier output hidden from the subject. Subjects were not informed that the presented feedback was biased until they had completed their last session.

The BCI system employed in this study is described in detail in [7]. Briey, classification was performed by logistic regression with *l*_1_-regularization, using logarithmic bandpower in frequency bands ranging from 7 to 40 Hz. Before bandpower computation, the 128-channel EEG data was spatially filtered using beamforming [7] (subjects 1 to 7 and 11) or Common Spatial Patterns (CSP) [8] (subjects 8 to 10).

**Figure 2:**
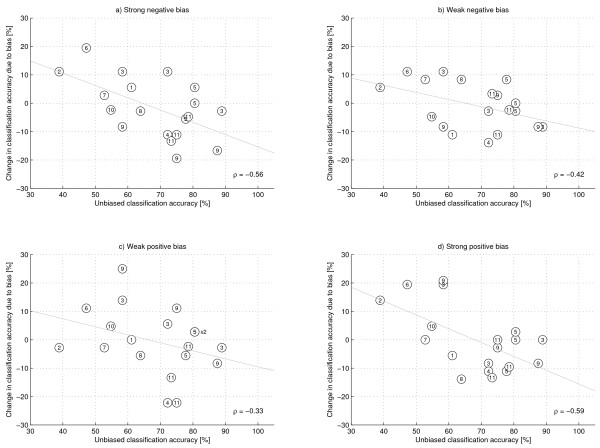
**Unbiased classification accuracy vs. deviation in accuracy due to feedback bias**. Unbiased classification accuracy vs. deviation from this accuracy due to feedback bias. A +10% value in the y-axis represents a 10% improvement in absolute mean accuracy. Each dot corresponds to one session, the numbers identificating the subjects. Least squares regression lines for each type of feedback bias are shown in grey along with their correlation coefficient. The x2 maker denotes overlapping datapoints corresponding to the same subject.

Mean classification accuracies across all subjects and sessions are shown in Table 1. While subject-specific effects of feedback bias could be observed (not shown here), mean classification accuracy was found to be around 68% for each type of feedback bias. In agreement with previous studies, this appears to indicate that the specific type of feedback had no general effect on BCI performance. However, Figure 2 shows the change in classification accuracy within a session due to each type of bias relative to the no-bias condition, with each dot representing one session and different subjects coded by number. Interestingly, for each type of feedback bias a negative correlation between unbiased classification accuracy and change in classification accuracy due to the bias could be observed. This correlation was found to be highly significant for a strong positive or negative bias (*p*_++ _= 0.0045, *p*_- - _= 0.0057), and only close to or weakly significant for a weak positive or negative bias (*p*_+ _= 0.0762, *p*_- _= 0.0384). All *p*-values were computed by random permutation analysis with 10,000 permutations and *n *= 648. Furthermore, the points of intersection of the regression lines in Figure 2 with zero change in classification accuracy roughly coincide with the unbiased classification accuracy required to reject chance-level classification accuracy (for each session, an accuracy of 63.9% is required to reject the null-hypothesis of chance-level classification accuracy at significance level *α *= 0.05). Our results hence appear to indicate that capable subjects, i.e., those with good classification accuracy without feedback bias, performed worse when given inaccurate feedback. Incapable subjects on the other hand, i.e., those that performed around chance-level, appeared to benefit from a feedback distortion. While it is not surprising that inaccurate feedback decreases performance for able subjects, an increase in classification accuracy due to a feedback bias in bad-performing subjects appears counterintuitive. To further probe this result, we computed mean classification accuracies with and without feedback-bias across all sessions for which the regression analysis suggested a beneficial effect of feedback bias, i.e, for sessions on the left hand side of the intersection of the regression line with zero-change in classification accuracy in Figure 2. This resulted in mean classification accuracies of 54.41% for the unbiased case, and 58.98%, 56.94%, 59.87%, and 61.78% for a strong negative, a weak negative, a weak postitive, and a strong positive bias, respectively (*n *= 256 for each type of bias). Using a binomial distribution, these classification accuracies were found to be sufficient for rejecting the null-hypothesis of chance-level performance at significance level *α *= 0.05 for a strong positive bias (*p *= 0.0003) as well as for a weak positive and strong negative bias (*p *= 0.0035 and *p *= 0.0120, respectively), but not for the unbiased case (*p *= 0.3761) and a weak negative bias (*p *= 0.0713) (Bonferroni correction for multiple comparisons).

**Table 1:** Mean classification results

Feedback bias	Classification accuracy
Strong positive bias (++)	68.06%

Weak positive bias (+)	67.44%

No bias	68.21%

Weak negative bias (-)	67.90%

Strong negative bias (- -)	66.82%

Mean classification accuracies across all subjects and sessions for each type of feedback bias.

As the study design required trials with different types of feedback to be interleaved as well as subjects remaining ignorant of the feedback distortion, we could not ask subjects to report their experiences regarding different types of feedback. As such, any interpretation of the observed effects currently remains speculative. We hypothesize that subjects already capable of utilizing a BCI for means of communication are able to make use of instantaneous and accurate feedback in order to optimally regulate their SMR. In these subjects, any type of feedback bias appears to interfere with this feedback loop and hence leads to degraded performance. Accurate feedback in incapable subjects, on the other hand, may be perceived as random noise, as the horizontal movement of the falling ball is uncorrelated with the intended movement direction. We hypothesize that this perceived lack of control leads to frustration and demotivation, impeding an effective skill acquisition process. In these subjects, biased feedback may reduce the perceived randomness of the visual feedback. Specifically, our results indicate that a strong positive bias may be particularly helpful for focussing on the intended task.

In terms of feedback design for future BCI systems, our results suggest that a subject's current skill level should be taken into account. Subjects already capable of modulating their sensorimotor rhythm to some extent should receive accurate feedback. Subjects not yet capable of utilizing a BCI, on the other hand, may benefit by designs that aim to induce a beneficial state-of-mind. While further investigations into the behavioral and neural correlates of a beneficial state-of-mind for BCIs are required (cf. [9,10] for two recent studies on this topic), the results presented here suggest that incapable subjects may particularly benefit if their belief on the level of control over the BCI-system is positively biased.

## Competing interests

The authors declare that they have no competing interests.

## Authors' contributions

AB carried out the BCI experiments for this study, adapted the BCI system to include the feedback bias, performed the statistical analysis and participated in the writing of the manuscript. MGW conceived and supervised the study, and participated in the data acquisition, statistical analysis and writing of the manuscript. All authors read and approved the final manuscript.
